# White matter microstructure is differently associated with executive functioning in youth born with congenital heart disease and youth born preterm

**DOI:** 10.1002/brb3.3308

**Published:** 2023-11-23

**Authors:** Kaitlyn Easson, May Khairy, Charles V. Rohlicek, Guillaume Gilbert, Annette Majnemer, Kim‐Anh Nguyen, Thuy Mai Luu, Élise Couture, Anne‐Monique Nuyt, Sean C. L. Deoni, Maxime Descoteaux, Marie Brossard‐Racine

**Affiliations:** ^1^ Advances in Brain & Child Development (ABCD) Research Laboratory Research Institute of the McGill University Health Centre Montreal Quebec Canada; ^2^ Department of Neurology & Neurosurgery, Faculty of Medicine & Health Sciences McGill University Montreal Quebec Canada; ^3^ Department of Pediatrics, Division of Neonatology Montreal Children's Hospital Montreal Quebec Canada; ^4^ Department of Pediatrics, Division of Cardiology Montreal Children's Hospital Montreal Quebec Canada; ^5^ MR Clinical Science Philips Healthcare Mississauga Ontario Canada; ^6^ School of Physical & Occupational Therapy, Faculty of Medicine & Health Sciences McGill University Montreal Quebec Canada; ^7^ Department of Pediatrics, Division of Neurology Montreal Children's Hospital Montreal Quebec Canada; ^8^ Department of Pediatrics, Division of Neonatology Jewish General Hospital Montreal Quebec Canada; ^9^ Department of Pediatrics Centre Hospitalier Universitaire Sainte‐Justine Montreal Quebec Canada; ^10^ Advanced Baby Imaging Lab Brown University Providence Rhode Island USA; ^11^ Sherbrooke Connectivity Imaging Laboratory (SCIL) Université de Sherbrooke Sherbrooke Quebec Canada; ^12^ Imeka Solutions Inc. Sherbrooke Quebec Canada

**Keywords:** congenital heart disease, neuropsychological functioning, preterm birth, white matter

## Abstract

**Introduction:**

Executive function deficits and adverse psychological outcomes are common in youth with congenital heart disease (CHD) or born preterm. Association white matter bundles play a critical role in higher order cognitive and emotional functions and alterations to their microstructural organization may result in adverse neuropsychological functioning. This study aimed to examine the relationship of myelination and axon density and orientation alterations within association bundles with executive functioning, psychosocial well‐being, and resilience in youth with CHD or born preterm.

**Methods:**

Youth aged 16 to 26 years born with complex CHD or preterm at ≤33 weeks of gestational age and healthy controls completed a brain MRI and self‐report assessments of executive functioning, psychosocial well‐being, and resilience. Multicomponent driven equilibrium single‐pulse observation of *T*_1_ and *T*_2_ and neurite orientation dispersion and density imaging were used to calculate average myelin water fraction (MWF), neurite density index (NDI), and orientation dispersion index values for eight bilateral association bundles. The relationships of bundle‐average metrics with neuropsychological outcomes were explored with linear regression and mediation analyses.

**Results:**

In the CHD group, lower MWF in several bundles was associated with poorer working memory and behavioral self‐monitoring and mediated self‐monitoring deficits relative to controls. In the preterm group, lower NDI in several bundles was associated with poorer emotional control and lower MWF in the left superior longitudinal fasciculus III mediated planning/organizing deficits relative to controls. No significant relationships were observed for psychosocial well‐being or resilience.

**Conclusion:**

The findings of this study suggest that microstructural alterations to association bundles, including lower myelination and axon density, have different relationships with executive functioning in youth with CHD and youth born preterm. Future studies should aim to characterize other neurobiological, social, and environmental influences that may interact with white matter microstructure and neuropsychological functioning in these at‐risk individuals.

## Graphical abstract

Microstructure in association bundles is differently related to executive functioning in youth born with congenital heart disease and youth born preterm. Microstructure in association bundles is not associated with psychosocial well‐being or resilience in youth born with congenital heart disease or born preterm.

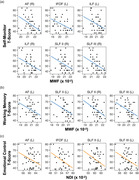

## 1 INTRODUCTION

Survivors of complex congenital heart disease (CHD) and preterm birth are both at high risk for neuropsychological impairments, which include various cognitive, behavioral, and emotional difficulties (Allen, 2008; Easson et al., 2019; Latal, 2016). Certain domains of neuropsychological functioning may become particularly relevant in adolescence and young adulthood as these individuals navigate the transition into independent adult life. For instance, executive functions are particularly important during this critical transitional period, linked to school and employment success, social functioning, quality of life, and mental health (Diamond, 2013). Indeed, executive function deficits are common in adolescents and young adults born with CHD (Bellinger et al., 2015; Ilardi et al., 2017) or born preterm (Burnett et al., 2015; Kroll et al., 2017). In addition, adverse psychological outcomes, such as poor psychosocial well‐being and resilience, may impact the ability of youth born with CHD or born preterm to navigate daily life, maintain social relationships, and adapt to challenges (Abda et al., 2019; Hack et al., 2007; Lee et al., 2014). Unfortunately, the neural correlates of these critical neuropsychological functions in these at‐risk youth remain poorly understood.

Association white matter bundles form the anatomical connections between distributed ipsilateral cortical regions and play an important role in higher order cognitive and emotional functions (Schmahmann et al., 2008). As such, microstructural alterations to these bundles may result in adverse neuropsychological functioning. We have previously used multicomponent driven equilibrium single‐pulse observation of *T*_1_ and *T*_2_ (mcDESPOT) (Deoni et al., 2013) and neurite orientation dispersion and density imaging (NODDI) (Zhang et al., 2012) to detect lower myelination and axon density, as measured by the myelin water fraction (MWF) and the neurite density index (NDI), respectively, in association bundles in youth born with CHD or born preterm, accompanied by alterations to axon orientation, as measured by the orientation dispersion index (ODI), in preterm‐born youth only (Easson et al., 2023).

Although previous studies have reported associations of MWF with cognitive development in healthy, term‐born infants and children (Deoni et al., 2013; O'Muircheartaigh et al., 2014) and of NDI and ODI with outcomes such as intelligence, processing speed, and behavioral/emotional problems in young children born preterm (Kelly et al., 2016; Sato et al., 2021; Young et al., 2019), no existing studies have examined the neuropsychological correlates of MWF, NDI, or ODI in adolescents or young adults with CHD or born preterm. As such, the impact of myelination, axon density, and axon orientation alterations on the neuropsychological functioning of youth with CHD or born preterm remains to be determined. Given the similarities of the neuropsychological profiles and white matter alterations of youth born with CHD or born preterm, there is value in exploring these structure–function relationships concurrently in these two populations.

Therefore, the objective of this study was to examine and compare the relationships of alterations to association bundle microstructure with neuropsychological functioning in youth born with CHD and youth born preterm. To accomplish this, we first evaluated if individual differences in association bundle microstructure are associated with variability in neuropsychological functioning among youth born with CHD and among youth born preterm. Afterwards, we investigated whether alterations in association bundle microstructure mediate neuropsychological deficits in the CHD and preterm groups relative to healthy controls.

## 2 MATERIALS AND METHODS

### 2.1 Participants

English‐ and French‐speaking youth aged 16 to 26 years born with complex CHD requiring open‐heart surgery before 2 years of age or born preterm at ≤33 weeks of gestational age without complex CHD, as well as a group of healthy controls, were recruited for this cross‐sectional study. Detailed inclusion and exclusion criteria and recruitment procedures have been previously described (Easson et al., 2023). Informed written consent was provided by participants aged 18 years and older and by the parents or legal guardians of participants younger than 18 years. This study was approved by the Pediatric Research Ethics Board of the McGill University Health Centre.

### 2.2 Assessments and questionnaires

Enrollees completed three self‐report questionnaires to assess executive functioning, psychosocial well‐being, and resilience. Executive functioning was assessed using the Behavior Rating Inventory of Executive Function—Adult Version (BRIEF‐A), a 75‐item self‐report questionnaire that assesses an individual's behavioral regulation and metacognition in their daily environment (Roth & Gioia, 2005). Scoring of the BRIEF‐A provides *T*‐scores, whereby higher *T*‐scores indicate worse functioning, on nine subscales (Inhibit, Shift, Emotional Control, Self‐Monitor, Initiate, Working Memory, Plan/Organize, Task Monitor, and Organization of Materials) and three summary indices (Behavioral Regulation Index, Metacognition Index, and Global Executive Composite).

Psychosocial well‐being was assessed using the Flourishing Scale, an 8‐item self‐report questionnaire of self‐perceived psychosocial success as related to self‐esteem, relationships, optimism, and sense of purpose (Diener et al., 2010). Total scores on the Flourishing Scale range from 8 to 56, with higher scores reflecting stronger psychosocial well‐being.

Resilience was assessed using the Resilience Scale, a 25‐item self‐report questionnaire that measures resilience as related to equanimity, perseverance, self‐reliance, meaningfulness, and existential aloneness (Wagnild & Young, 1993). Total scores on the Resilience Scale range from 25 to 175, with higher scores reflecting stronger resilience. Of note, the Resilience Scale was added to our study protocol partway through data collection. Responses for this questionnaire were collected where possible through an online questionnaire from participants who had already completed their in‐person study visit.

Participants also completed in‐house questionnaires to collect demographic and individual characteristics. Maternal education and employment were measured on 7‐point and 9‐point scales (Table S1), respectively, based on the Hollingshead Four‐Factor Index (Hollingshead, 2011). Maternal education was selected as our measure of socioeconomic status, as it is one of the strongest predictors of cognitive and emotional development (Harding et al., 2015; Maggi et al., 2010). One missing maternal education score was imputed as the mean score among mothers with the same employment level. Together, the self‐report assessments and in‐house questionnaires took approximately 30 minutes to complete.

### 2.3 MRI acquisition and processing

Our MRI acquisition and processing protocols have been previously described elsewhere (Easson et al., 2022, 2023, 2020). Participants completed a brain MRI on a 3T MRI system, including a *T*_1_‐weighted anatomical image, a high angular resolution diffusion imaging (HARDI) acquisition comprised of a non‐diffusion weighted volume with reverse phase encoding and two single‐shell HARDI sequences (*b* = 700 s/mm^2^ and 30 directions; *b* = 2000 s/mm^2^ and 60 directions), and a mcDESPOT acquisition comprised of 10 spoiled gradient recalled echo (SPGR) sequences, 8 balanced steady‐state free precession sequences with 0° and 180° phase‐cycling, and an inversion recovery SPGR sequence. Relevant acquisition parameters are detailed in Table S2. In total, the duration of these sequences was approximately 36 minutes.

In brief, our data processing protocol included: preprocessing and probabilistic particle filter tractography with the TractoFlow pipeline (Di Tommaso et al., 2017; Kurtzer et al., 2017; Theaud et al., 2020); mcDESPOT modeling (Deoni et al., 2013); NODDI modeling (Daducci et al., 2015; Zhang et al., 2012) with custom parallel and isotropic diffusivity priors of approximately 1.64 × 10^−3^ and 3.33 × 10^−3^ mm^2^/s (Easson et al., 2020); bundle extraction with a modified version of RecoBundles (Garyfallidis et al., 2018); and tractometry (Cousineau et al., 2017) to calculate average values of MWF, NDI, and ODI for eight bilateral association bundles. The association bundles of interest were as follows: the left and right arcuate fasciculus, cingulum, inferior frontal occipital fasciculus, inferior longitudinal fasciculus, uncinate fasciculus, and superior longitudinal fasciculus I, II, and III. Participants or individual white matter bundles who failed visual quality inspection were excluded from analysis.

### 2.4 Statistical analysis

Descriptive statistics were used to characterize the groups in terms of their individual characteristics and outcomes. *F*‐tests and post hoc Tukey's tests were used to compare continuous variables, whereas *χ* ^2^ tests and Bonferroni post hoc testing of *χ* ^2^ residuals were used to compare categorical variables between groups. Neuropsychological outcomes were compared between groups in a pairwise fashion with separate linear models controlling for age, sex, and maternal education score. These analyses were exploratory and were not adjusted for multiple comparisons.

Associations between bundle‐average MWF, NDI, and ODI of the eight bilateral association bundles and all neuropsychological outcomes were first explored with a series of multiple linear regression models, fit separately in the three groups, and controlling for age, sex, and maternal education score as covariates. Bundle‐average metric values were rescaled to units of 0.01 to improve interpretability of unstandardized regression coefficients. The false discovery rate method was used to correct for multiple comparisons across white matter bundles, with a threshold of statistical significance of *q* < .05.

**FIGURE 1:**
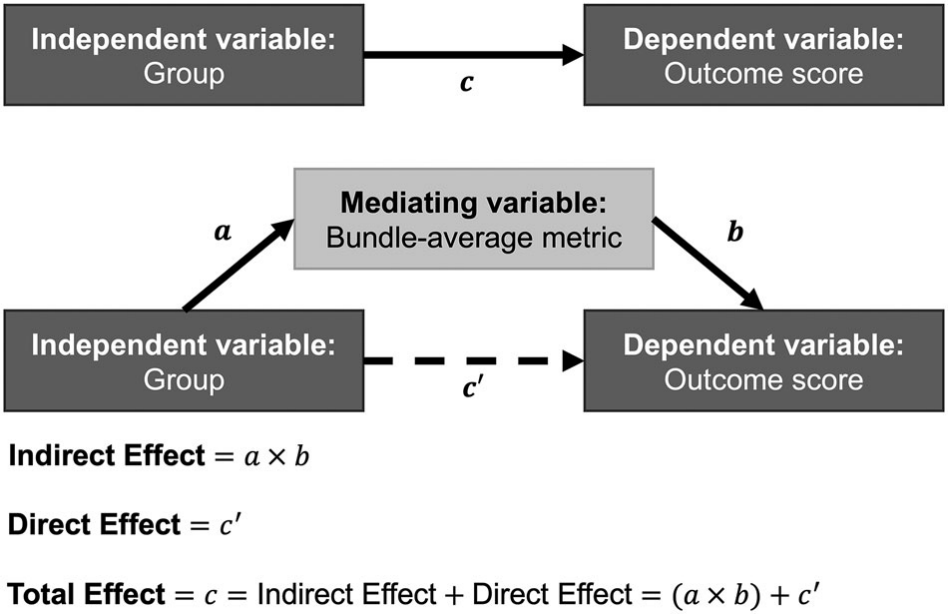
Visualization of mediation model and metrics of interest. All models controlled for age, sex, and maternal education score as covariates.

Afterward, mediation analyses were performed for combinations of outcomes where the CHD or preterm group had significantly poorer performance as compared to the control group and bundle‐average metrics we have previously shown to be significantly different in the CHD or preterm group as compared to controls (Easson et al., 2023). For these models, outcome score was the dependent variable, group (CHD vs. control or preterm vs. control) was the independent variable, and bundle‐average MWF, NDI, or ODI of a specific bundle was the mediating variable (Figure 1). Age, sex, and maternal education score were controlled for as covariates. In each mediation model, the total effect, representing the overall effect of group on an outcome score, is partitioned into indirect and direct effects. The indirect effect represents the component of the total effect of group on an outcome score that is mediated by a particular bundle‐average metric. The direct effect represents the remaining, non‐mediated component of the total effect. Bootstrapping with 1000 simulations was used to test for statistical significance and compute bias‐corrected and accelerated confidence intervals (DiCiccio & Efron, 1996). Given the exploratory nature of these analyses, multiple comparison correction was not applied. We considered a significant mediation effect to occur when the indirect effect was significant at *p* < .05. All statistical analyses were performed in R version 3.6.3, leveraging the *mediation* package for mediation analyses and the *ggplot2* package for data visualization.

## 3 RESULTS

### 3.1 Participant characteristics

A total of 164 participants (53 youth born with CHD, 55 youth born preterm, and 56 healthy controls) were enrolled in the study. One participant in each group did not complete all the acquisitions required for the current analyses, whereas 8 CHD participants, 10 preterm participants, and 8 control participants were excluded after failing quality inspection of MRI data. Excluded participants did not differ significantly from included participants in terms of their demographic characteristics. The characteristics of the final sample of 44 CHD, 44 preterm, and 47 control participants are outlined in Table 1. There was a significant effect of group on maternal education, driven by higher maternal education scores in the control group as compared to the CHD (*p* < .001) and preterm (*p* = .006) groups. There was also a significant effect of group on participants’ current educational enrollment, driven by a higher proportion of currently enrolled students in the control group (*p* = .049). Clinical characteristics of the CHD and preterm groups are outlined in Table 2. Overt brain abnormalities in the study sample, as detected with conventional MRI, have previously been described in detail and were found to not be significantly associated with MWF, NDI, or ODI (Easson et al., 2023).

**TABLE 1:** Participant characteristics.

	CHD (*N* = 44)	Preterm (*N* = 44)	Control (*N* = 47)	*p* Value
**Age at MRI (years)**	19.9 ± 2.3	20.2 ± 3.1	20.7 ± 2.5	.368
**Sex**				.778
Female	25 (56.8%)	24 (54.5%)	29 (61.7%)	
Male	19 (43.2%)	20 (45.5%)	18 (38.3%)	
**Maternal education score**	5.2 ± 1.3	5.4 ± 1.2	6.1 ± 1.1	**<.001**
**Currently employed**	23 (53.5%)	28 (63.6%)	26 (55.3%)	.591
**Currently a student**	33 (75.0%)	36 (81.8%)	45 (95.7%)	**.020**

*Note*: Data are presented as mean ± SD for continuous variables and n (%) for binary variables.

Abbreviation: CHD, congenital heart disease.

**TABLE 2:** Clinical characteristics of congenital heart disease (CHD) and preterm groups.

*CHD group*	
**Two‐ventricle physiology**	37 (84.1%)
Dextro‐transposition of the great arteries	15 (34.1%)
Tetralogy of Fallot	12 (27.3%)
Ventricular septal defect	4 (9.1%)
Double outlet right ventricle	2 (4.5%)
Total anomalous pulmonary venous connection	2 (4.5%)
Ebstein's anomaly	1 (2.3%)
Truncus arteriosus type I	1 (2.3%)
**Single‐ventricle physiology**	7 (15.9%)
Pulmonary atresia with intact ventricular septum	3 (6.8%)
Double inlet left ventricle	2 (4.5%)
Hypoplastic left heart syndrome	1 (2.3%)
Tricuspid atresia	1 (2.3%)
**Number of open‐heart surgeries**	1 [1–4]
**Age at first open‐heart surgery (days)**	35 [0–702]

*Preterm group*	
**Gestational age at birth (weeks)**	28.1 ± 2.2
**Birth weight (grams)**	970 ± 268

*Note*: Data are presented as mean ± SD or median [range] for continuous variables and *n* (%) for categorical variables.

### 3.2 Neuropsychological outcomes

**FIGURE 2:**
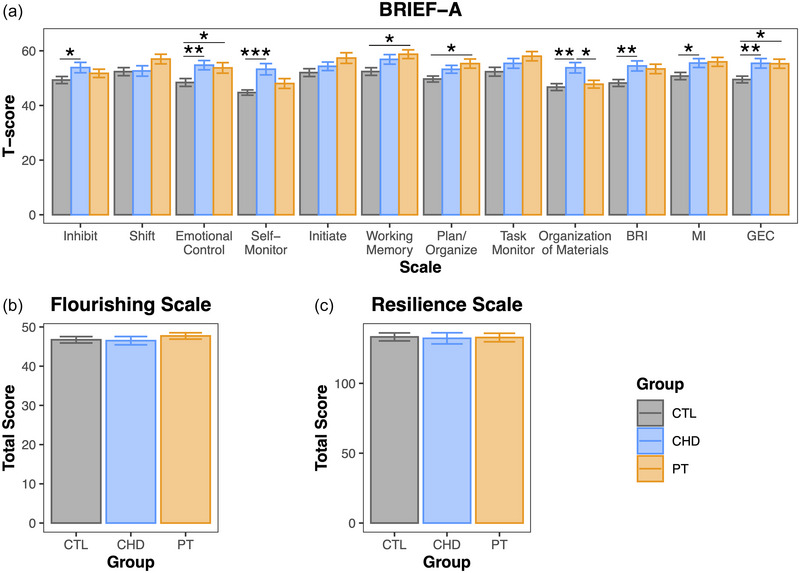
Group comparisons of neuropsychological outcomes: (a) comparisons of BRIEF‐A *T*‐scores (Control: *N* = 46; CHD: *N* = 43; PT: *N* = 44); (b) comparison of Flourishing Scale total scores (Control: *N* = 47; CHD: *N* = 44; PT: *N* = 44); (c) comparison of Resilience Scale total scores (Control: *N* = 32; CHD: *N* = 25; PT: *N* = 44). *p*‐Values computed from pairwise group comparisons with linear models controlling for age, sex, and maternal education score. Data are presented as mean ± standard error. *p < .05; **p < .01; ***p < .001. BRI, Behavioral Regulation Index; BRIEF‐A, Behavior Rating Inventory of Executive Function—Adult Version; CHD, congenital heart disease; CTL, control; GEC, Global Executive Composite; MI, Metacognition Index, PT, preterm.

With respect to the BRIEF‐A (Figure 2a), the CHD group presented with significantly higher *T*‐scores than the control group, indicating poorer functioning, on the Inhibit, Emotional Control, Self‐Monitor, and Organization of Materials subscales, as well as on the Behavioral Regulation Index, Metacognition Index, and Global Executive Composite. The preterm group presented with significantly higher *T*‐scores than the control group on the Emotional Control, Working Memory, and Plan/Organize subscales, as well as on the Global Executive Composite. The CHD and preterm groups only differed significantly on the Organization of Materials subscale, with higher *T*‐scores in the CHD group. No group differences were detected for the Flourishing Scale (Figure 2b) or Resilience Scale (Figure 2c).

### 3.3 Structure–function linear regression analyses

**FIGURE 3:**
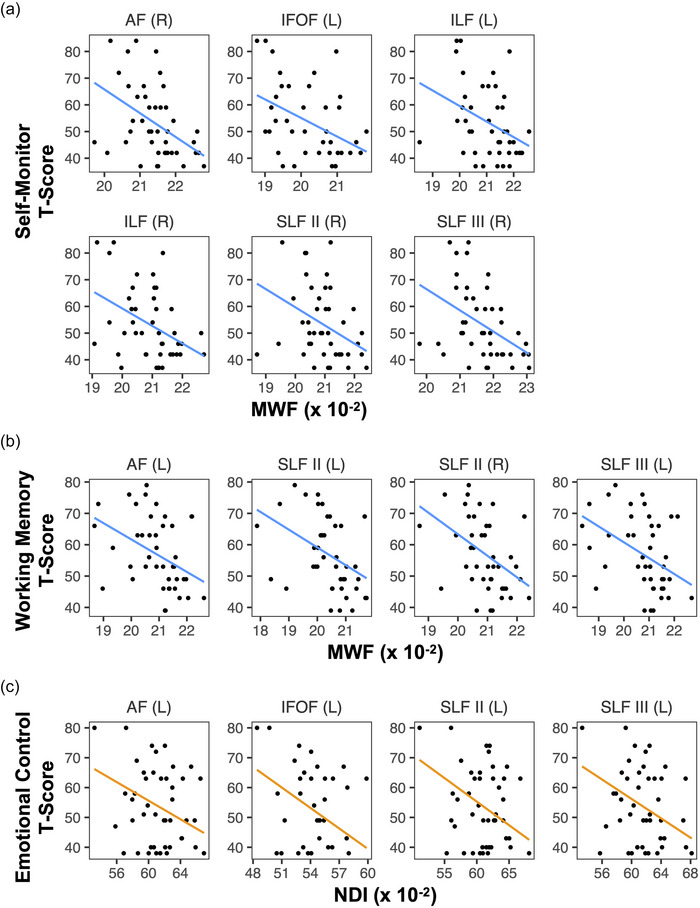
Scatterplots of significant linear regression results: (a) associations between myelin water fraction (MWF) and self‐monitor *T*‐scores in the congenital heart disease (CHD) group; (b) associations between MWF and working memory *T*‐scores in the CHD group; (c) associations between neurite density index (NDI) and emotional control *T*‐scores in the preterm group. Colored lines represent the corresponding regression line, evaluated at average covariate values. AF, arcuate fasciculus; IFOF, inferior frontal occipital fasciculus; ILF, inferior longitudinal fasciculus; SLF, superior longitudinal fasciculus.

Several different significant associations between bundle‐average MRI metrics and BRIEF‐A *T*‐scores were detected in the CHD and preterm groups, but none were found in the control group (Table 3). In the CHD group, negative associations between bundle‐average MWF and BRIEF‐A Self‐Monitor *T*‐scores were found in the right arcuate fasciculus, left inferior frontal occipital fasciculus, bilateral inferior longitudinal fasciculus, and right superior longitudinal fasciculus II and III (Figure 3a). Negative associations between bundle‐average MWF and BRIEF‐A Working Memory *T*‐scores were found in the left arcuate fasciculus, bilateral superior longitudinal fasciculus II, and left superior longitudinal fasciculus III (Figure 3b). In the preterm group, negative associations between bundle‐average NDI and BRIEF‐A Emotional Control *T*‐scores were found in the left arcuate fasciculus, inferior frontal occipital fasciculus, and superior longitudinal fasciculus II and III (Figure 3c). No significant associations between MWF, NDI, or ODI and scores on the Flourishing Scale or Resilience Scale were found in any of the groups.

**TABLE 3:** Significant linear regression results.

*CHD group*						
Outcome	Metric	Bundle	*β* [95% CI]	*β*_standardized_	*q* Value	*R* ^2^_adj_
Self‐monitor	MWF^a^	AF (R)	−8.89 [−14.52, −3.27]	−0.460	.032	.214
		IFOF (L)	−6.98 [−12.00, −1.96]	−0.424	.032	.162
		ILF (L)	−5.82 [−10.60, −1.05]	−0.371	.048	.141
		ILF (R)	−6.56 [−11.29, −1.82]	−0.409	.032	.174
		SLF II (R)	−6.81 [−12.15, −1.47]	−0.387	.044	.151
		SLF III (R)	−7.94 [−13.32, −2.56]	−0.439	.032	.192
Working memory	MWF^a^	AF (L)	−5.19 [−9.02, −1.37]	−0.406	.037	.146
		SLF II (L)	−5.67 [−9.52, −1.81]	−0.431	.030	.167
		SLF II (R)	−6.88 [−11.31, −2.45]	−0.462	.030	.188
		SLF III (L)	−5.05 [−8.54, −1.57]	−0.439	.030	.166

*Preterm group*						
Outcome	Metric	Bundle	*β* [95% CI]	*β*_standardized_	*q* Value	*R* ^2^_adj_
Emotional control	NDI^a^	AF (L)	−1.56 [−2.75, −0.37]	−0.364	.047	.249
		IFOF (L)	−2.29 [−3.94, −0.63]	−0.442	.045	.315
		SLF II (L)	−1.58 [−2.65, −0.50]	−0.410	.045	.278
		SLF III (L)	−1.62 [−2.78, −0.47]	−0.393	.045	.267

*Note*: Models corrected for age, sex, and maternal education score. Only significant associations between MRI metrics and outcome scores at *q* < .05 are displayed. Unstandardized (*β*) and standardized (*β*_standardized_) regression coefficients, adjusted for covariates, are presented. *R* ^2^_adj_ values are provided to describe the overall fit of the full model, including covariates.

Abbreviations: AF, arcuate fasciculus; CI, confidence interval; CHD, congenital heart disease IFOF, inferior frontal occipital fasciculus; ILF, inferior longitudinal fasciculus; MWF, myelin water fraction; NDI, neurite density index; SLF, superior longitudinal fasciculus.

^a^ Metric values rescaled to units of 0.01 to improve interpretability of unstandardized regression coefficients.

### 3.4 Structure–function mediation analyses

Lower bundle‐average MWF relative to controls mediated poorer performance on different BRIEF‐A subscales in the CHD and preterm groups (Table 4). In the CHD group, higher *T*‐scores on the Self‐Monitor subscale relative to controls were mediated by lower MWF in the bilateral arcuate fasciculus, left inferior frontal occipital fasciculus, right inferior longitudinal fasciculus, left superior longitudinal fasciculus I, left superior longitudinal fasciculus II, and bilateral superior longitudinal fasciculus III. In addition, higher *T*‐scores on the Behavioral Regulation Index and Global Executive Composite were mediated by lower MWF in the left superior longitudinal fasciculus I and higher *T*‐scores on the Metacognition Index were mediated by lower MWF in the left superior longitudinal fasciculus III. In the preterm group, higher *T*‐scores on the Plan/Organize subscale relative to controls were mediated by lower MWF in the left superior longitudinal fasciculus III only.

**TABLE 4:** Significant mediation analysis results.

*CHD group*					
Outcome	Metric	Bundle	Indirect effect [95% BCa CI]	Direct effect [95% BCa CI]	Total effect [95% BCa CI]
Self‐monitor	MWF	AF (L)	2.19 [0.57, 5.38]*	6.47 [1.58, 11.46]**	8.66 [4.14, 13.59]***
		AF (R)	1.79 [0.39, 4.97]*	6.86 [2.04, 11.45]**	8.66 [4.14, 13.59]***
		IFOF (L)	2.61 [0.18, 7.23]*	5.67 [0.04, 11.12]	8.28 [3.40, 13.59]**
		ILF (R)	1.88 [0.24, 4.99]*	6.77 [2.20, 11.43]**	8.66 [4.14, 13.59]***
		SLF I (L)	2.41 [0.80, 5.73]**	6.25 [0.85, 10.57]**	8.66 [4.14, 13.59]***
		SLF II (L)	2.76 [1.07, 5.85]**	5.90 [0.96, 10.72] *	8.66 [4.14, 13.59]***
		SLF III (L)	1.90 [0.44, 5.28]*	6.75 [1.95, 11.57]**	8.66 [4.14, 13.59]***
		SLF III (R)	1.36 [0.27, 3.99]*	7.30 [2.43, 11.90]***	8.66 [4.14, 13.59]***
BRI	MWF	SLF I (L)	1.87 [0.24, 4.78]*	5.08 [−0.26, 9.90]	6.95 [2.01, 11.98]**
MI	MWF	SLF III (L)	2.09 [0.32, 4.79]*	2.45 [−2.30, 7.10]	4.54 [0.05, 8.86]*
GEC	MWF	SLF I (L)	2.06 [0.19, 5.15]*	4.24 [−0.87, 8.80]	6.31 [1.83, 10.70]**

*Preterm group*					
Outcome	Metric	Bundle	Indirect effect [95% BCa CI]	Direct effect [95% BCa CI]	Total effect [95% BCa CI]
Plan/Organize	MWF	SLF III (L)	1.85 [0.32, 4.83]*	3.22 [−1.30, 7.47]	5.07 [0.74, 9.28]*

*Note*: Models corrected for age, sex, and maternal education score. Only models with significant indirect effects at *p* < .05 are displayed.

Abbreviations: AF, arcuate fasciculus; BCa CI, bias‐corrected and accelerated confidence interval; BRI, Behavioral Regulation Index; GEC, Global Executive Composite; IFOF, inferior longitudinal fasciculus; ILF, inferior longitudinal fasciculus; MI, Metacognition Index; MWF, myelin water fraction; SLF, superior longitudinal fasciculus.

*p < .05; **p < .01; ***p < .001.

## 4 DISCUSSION

The findings of this study suggest that specific microstructural features in various association bundles are related to executive functioning, but not self‐reported psychosocial well‐being or resilience, in youth born with CHD and youth born preterm. Interestingly, despite presenting with a similar risk for executive dysfunction and alterations to myelination and axon density (Easson et al., 2023), the precise nature of the observed structure–function relationships differed across groups with respect to their microstructural elements and specific outcomes. Overall, our findings suggest that while alterations to white matter microstructure are related to executive functioning in youth born with CHD or born preterm, the neuropsychological difficulties experienced by these individuals are likely multifactorial in origin and as such, other biological, social, and environmental factors also need to be considered to obtain a more comprehensive understanding of their underlying mechanisms.

### 4.1 Microstructural correlates of executive functioning

There is considerable variability in neuropsychological functioning among youth born with CHD and among youth born preterm. Attempting to identify factors that might explain why some individuals have poorer outcomes, we first performed group‐stratified multiple linear regression analyses to examine if differences in white matter microstructure among youth born with CHD or among youth born preterm are related to within‐group variability in outcome. In the CHD group, these analyses revealed that lower myelination of several association bundles is related to poorer working memory and behavioral self‐monitoring. Myelination of superior longitudinal fasciculus subdivisions and of the arcuate fasciculus was found to be associated with both domains, aligning with previous evidence from diffusion tensor imaging (DTI) and lesion mapping studies linking these bundles to working memory and overall executive functioning (Barbey et al., 2012; Koshiyama et al., 2020; Lu et al., 2016; Short et al., 2013; Urger et al., 2015). In addition, myelination of the inferior longitudinal fasciculus and inferior frontal occipital fasciculus was linked to behavioral self‐monitoring only. These two bundles have been implicated in the recognition of facial emotions (Crespi et al., 2014; Genova et al., 2015; Philippi et al., 2009), which may contribute to successful behavioral self‐monitoring by allowing individuals to recognize their peers’ emotional responses to their behavior. Although to our knowledge, no previous study has evaluated the relationship between myelin‐specific metrics and neuropsychological functioning, our results align to some extent with previous DTI studies that reported associations between white matter fractional anisotropy and executive functioning in adolescents and adults born with CHD (Ehrler et al., 2020, 2021; Rollins et al., 2014). Our findings suggest that altered myelination may be a specific microstructural feature driving these previous observations.

In the preterm group, we observed that lower axon density in the left arcuate fasciculus, inferior frontal occipital fasciculus, and superior longitudinal fasciculus II and III was related to poorer emotional control. These findings are consistent with a previous NODDI study that uncovered negative associations between NDI in the superior longitudinal fasciculus and inferior frontal occipital fasciculus and behavioral/emotional problems in 7‐year‐old children born very preterm (Kelly et al., 2016), and broadly align with previous studies in other clinical populations that have suggested a role of the arcuate fasciculus in emotional regulation (David et al., 2020; Spitz et al., 2017).

Overall, across the significant linear regression results uncovered in the CHD and preterm groups, the observed ranges of standardized regression coefficients are indicative of moderate effect sizes. This is broadly in line with previous reports of relationships between DTI measures of white matter microstructure and executive functioning in these populations with effect sizes in a similar range (Allin et al., 2011; Ehrler et al., 2020, 2021).

In contrast, no significant relationships were detected between white matter microstructure and neuropsychological functioning in the control group. This aligns with previous DTI studies reporting significant structure–function relationships in youth with CHD or born preterm, but not in their associated control groups (Brewster et al., 2015; Eikenes et al., 2011; Vollmer et al., 2017; Watson et al., 2018). However, it is possible that our control group, comprised predominantly of currently enrolled students, does not capture the full range of neuropsychological functioning present in the general population, which may hinder the detection of significant structure–function relationships.

### 4.2 Mediation of executive function impairments by myelination deficits

At the group level, individuals born with CHD and individuals born preterm often present with significantly poorer performance relative to healthy controls across several neuropsychological domains. Mediation analyses can provide useful insight into the factors that may underlie these group differences. As such, as a second analytical approach, we performed mediation analyses to examine if poorer neuropsychological performance in the CHD or preterm groups as compared to controls is driven by group differences in white matter microstructural features. Notably, in both clinical groups, deficient myelination was the only microstructural alteration found to significantly mediate executive function difficulties relative to controls. This may be because, as we have previously reported, myelination deficits relative to controls are widespread and pronounced in both youth born with CHD or born preterm, whereas alterations to axon density or orientation are more limited (Easson et al., 2023).

In the CHD group, we observed that lower myelination in the superior and inferior longitudinal fasciculi, arcuate fasciculus, and inferior frontal occipital fasciculus mediated poorer behavioral self‐monitoring relative to the control group, closely mirroring our findings for this subscale from our linear regression analyses. Furthermore, lower myelination of superior longitudinal fasciculus subdivisions mediated deficits in overall behavioral regulation, metacognition, and global executive functioning in the CHD group relative to controls, suggesting a broader impact of lower myelination of this bundle on performance in this group. In the preterm group, the mediation analyses indicated that lower myelination of the superior longitudinal fasciculus also mediated executive function difficulties relative to controls, but for a different domain. Specifically, lower myelination of the superior longitudinal fasciculus III mediated poorer planning/organizing in the preterm group relative to controls. This observed mediation effect aligns with functional MRI work establishing the importance of frontal–parietal brain networks in autobiographical and future planning (Gerlach et al., 2014; Spreng et al., 2010).

### 4.3 Future directions

Our findings highlight that association bundle microstructure, and particularly myelination, may be an important factor for executive functioning in youth born with CHD or born preterm, and as such, should be considered in future work exploring the neural underpinnings of functional deficits compared to healthy peers. Nonetheless, it is unclear why the CHD and preterm groups presented with distinct structure–function relationships in the present study, despite their common propensity for executive function deficits and their previously reported similar patterns of altered myelination and axon density (Easson et al., 2023). This likely reflects the multifactorial complexity of investigations aiming to identify the determinants of functional outcomes. Indeed, beyond other neurobiological correlates, such as alterations to gray matter volumes (Fontes et al., 2019) or functional connectivity (Enguix et al., 2022), there may be numerous social and environmental influences, not captured in the present study, that are relevant for the neuropsychological development of survivors of CHD or preterm birth across the lifespan. These influences may be particularly relevant for psychosocial well‐being and resilience, for which we observed no relationships with association bundle microstructure. Indeed, extrinsic factors, such as parental mental health and distress, parenting style, and quality of the home environment, have been shown to influence cognitive and emotional development in children born with CHD or born preterm (Bonthrone et al., 2021; de Silva et al., 2021; Gupta et al., 1998; McCusker et al., 2007; Treyvaud et al., 2012; Visconti et al., 2002). Furthermore, previous work has shown that positive and negative social influences from peers affect emotional well‐being and resilience in typically developing adolescents and young adults (Kef & Dekovic, 2004; Lopez & Dubois, 2005; Van Geel Et Al., 2018; Wilks & Spivey, 2010). Future studies of survivors of CHD or preterm birth that incorporate multimodal neuroimaging with measures of social and environmental influences will be required to investigate the individual effects of these various factors and how they may interact with white matter microstructure to influence the neuropsychological development of these at‐risk individuals. This will facilitate a deeper understanding of the similarities and differences in the etiology of the neuropsychological difficulties commonly experienced by individuals born with CHD or born preterm. As a component of this work, emphasis should be placed on identifying modifiable risk and protective factors that may provide an avenue for intervention to promote optimal neuropsychological development.

### 4.4 Limitations

The main limitation of this study is our exclusive use of self‐report questionnaires to measure the outcomes of interest, which may have allowed for the introduction of response bias. Although the chosen measures have demonstrated their reliability and validity in various groups (Ciszewski et al., 2014; De La Fuente Et Al., 2017; Humphreys, 2003; Rabin et al., 2006), performance‐based evaluations of executive functioning and informant‐reports of psychological outcomes may have provided more objective measurements. Moreover, we examined a large number of combinations of MRI metrics, association bundles, and neuropsychological outcomes, which may have resulted in spurious findings. In addition, we were missing a considerable amount of data for the Resilience Scale in the CHD and control groups, which may have limited our statistical power to detect significant associations between resilience and white matter microstructure.

Furthermore, the use of bundle‐average measures of MWF, NDI, and ODI may have prevented us from detecting more subtle, spatially limited associations between neuropsychological functioning and white matter microstructure that could be detected with techniques such as tract‐based spatial statistics. However, our approach allowed us to focus on clinically relevant associations that were pronounced enough to be detected at the whole‐bundle level. Additionally, MWF, NDI, and ODI are indirect estimates of myelination, axon density, and axon orientation and should not be considered direct measurements of these microstructural elements. Finally, our two clinical groups were relatively heterogeneous, which may limit the generalizability of our findings to specific subgroups of CHD or preterm birth survivors.

## 5 CONCLUSION

In conclusion, this study provides evidence that altered association bundle microstructure, specifically involving lower myelination or axon density, is differently associated with executive dysfunction in unique ways in youth born with CHD and youth born preterm. In contrast, no significant relationships were detected between white matter microstructure and psychosocial well‐being or resilience. Future work should aim to construct comprehensive models of the various neurobiological, social, and environmental factors that may interact with one another to influence trajectories of neuropsychological development in survivors of CHD or preterm birth through adolescence into independent adult life.

## AUTHOR CONTRIBUTIONS

**Kaitlyn Easson**: Conceptualization; data curation; formal analysis; investigation; project administration; visualization; writing—original draft; writing—review and editing. **May Khairy**: Investigation; resources; writing—review and editing. **Charles V. Rohlicek**: Investigation; resources; writing—review and editing. **Guillaume Gilbert**: Methodology; software; writing—review and editing. **Sean C. L. Deoni**: Methodology; software; writing—review and editing. **Annette Majnemer**: Resources; writing—review and editing. **Kim‐Anh Nguyen**: Resources; writing—review and editing. **Thuy Mai Luu**: Resources; writing—review and editing. **Élise Couture**: Resources; writing—review and editing. **Anne‐Monique Nuyt**: Resources; writing—review and editing. **Maxime Descoteaux**: Methodology; software; supervision; writing—review and editing. **Marie Brossard‐Racine**: Conceptualization; funding acquisition; investigation; methodology; project administration; resources; supervision; writing—original draft; writing—review and editing.

## CONFLICT OF INTEREST STATEMENT

G. Gilbert is an employee of Philips Healthcare Canada. M. Descoteaux is an employee, cofounder, and co‐owner of Imeka Solutions Inc. The authors have no other interests to disclose.

### PATIENT CONSENT STATEMENT

Informed written consent was provided by participants aged 18 years and older and by the parents or legal guardians of participants younger than 18 years.

### PEER REVIEW

The peer review history for this article is available at https://publons.com/publon/10.1002/brb3.3308.

## Supporting information


**Table S1** Maternal education and employment scoring schemes.
**Table S2** MRI acquisition parameters.

## Data Availability

The data that support the findings of this study are available from the corresponding author upon reasonable request.
