# Supplementary material for: White matter microstructure is differently associated with executive functioning in youth born with congenital heart disease and youth born preterm
Source: Brain Behav. 2023 Nov 23;13(12):e3308. doi: 10.1002/brb3.3308 (PMC10726855; doi:10.1002/brb3.3308)
Supplement: Supplementary file 1 — Table S1 Maternal education and employment scoring schemes. Table S2 MRI acquisition parameters. [file BRB3-13-e3308-s001.docx]

**Supplementary Material**

**Table S1.** Maternal education and employment scoring schemes.

| **Maternal education** | |
| --- | --- |
| 1 | 6^th^ grade or less. |
| 2 | 9^th^ grade completed. |
| 3 | 10^th^ grade completed. |
| 4 | High school completed. |
| 5 | CEGEP, college certification, or technical program completed. |
| 6 | Undergraduate degree completed. |
| 7 | Graduate or professional degree completed. |
| **Maternal employment** | |
| 1 | Farm laborer/Menial service workers (e.g., custodians, gardeners, dishwashers). |
| 2 | Unskilled workers (e.g., bartenders, cooks, food service, labourers). |
| 3 | Machine operators, semiskilled workers (e.g., truck drivers, assemblers, hairdressers). |
| 4 | Skilled manual workers, craftsmen (e.g., electricians, mechanics, receptionists). |
| 5 | Clerical and sales workers (e.g., bank tellers, cashiers, therapy assistants). |
| 6 | Technicians, semi-professionals (e.g., administrators, therapists, technicians). |
| 7 | Small business owners, minor professionals, managers (e.g., computer programmers,  real estate agents, sales managers). |
| 8 | Medium business owners, lesser professionals (e.g., accountants, pharmacists, registered  nurses). |
| 9 | Major business owners, higher professionals, higher executives (e.g., lawyers, doctors,  professors). |

*Abbreviations:* CEGEP = Collège d'enseignement général et professionnel.

**Table S2.** MRI acquisition parameters.

| **T1-Weighted** | **HARDI** | **mcDESPOT** |
| --- | --- | --- |
| - TR = 8.1 ms - TE = 3.7 ms - TI = 1010 ms - Flip angle = 8° - Voxel size = 1.00 x 1.00 x 1.00 mm^3^ | - TR = 9400 ms - TE = 78 ms - Flip angle = 90° - Voxel size = 2.00 x 2.04 x 2.00 mm^3^ | **SPGR sequences:**   - TR = 6.7 ms - TE = 3.7 ms - Flip angle range = 2° to 18° - Voxel size = 1.67 x 1.67 x 1.70 mm^3^   **bSSFP sequences:**   - TR = 6.8 ms - TE = 3.4 ms - Flip angle range = 12° to 70° - Voxel size = 1.67 x 1.67 x 1.70 mm^3^   **IR-bSSFP sequence:**   - TR = 6.5 ms - TE = 3.2 ms - TI = 450 ms - Flip angle = 5° - Voxel size = 1.67 x 1.67 x 1.70 mm^3^ |

*Abbreviations:* bSSFP = balanced steady-state free precession; HARDI = high angular resolution diffusion imaging; IR-SPGR = inversion recovery spoiled gradient recalled echo; mcDESPOT = multicomponent driven equilibrium single pulse observation of T_1_ and T_2_; SPGR = spoiled gradient recalled echo; TE = echo time; TI = inversion time; TR = repetition time.
